# Combination Treatment of Arazyme and Soy Leaf Extract Attenuates Hyperglycemia and Hepatic Steatosis in High-Fat Diet-Fed C57BL/6J Mice

**DOI:** 10.3390/life11070645

**Published:** 2021-07-01

**Authors:** Hwa Lee, Seona Cho, Anna Kang, Dong-Ha Shin, Ho-Yong Park, Tae-Sook Jeong

**Affiliations:** 1 Industrial Bio-Materials Research Center, Korea Research Institute of Bioscience and Biotechnology, Daejeon 34141, Korea; leehua@kribb.re.kr (H.L.); baby5624@naver.com (S.C.); ank4200@gmail.com (A.K.); 2 Insect Biotech Co., Ltd., Daejeon 34054, Korea; dhshin@insectbiotech.co.kr

**Keywords:** Arazyme, soy leaf, diet therapy, hepatic steatosis, hyperglycemia, obesity

## Abstract

Arazyme and extracts of soy leaves (ESLs) are used as ingredients for functional foods; however, their combined administration has not been studied. This study assessed the combined effect of Arazyme and ESLs in high-fat-diet (HFD)-induced obese C57BL/6J mice fed 2 mg/kg Arazyme, 50 mg/kg ESLs, or a combination of 2 mg/kg Arazyme and 50 mg/kg ESLs by oral gavage for 13 weeks. Individually, Arazyme and ESLs had no effect on the HFD-induced phenotypes. The combination of Arazyme and ESLs significantly suppressed body weight gain, improved glucose and insulin tolerance, and suppressed hepatic steatosis by reducing lipid synthesis and enhancing lipid utilization gene expression. Furthermore, the combination significantly reduced HFD-induced plasma bile acid reabsorption by suppressing bile acid transporter expression, including the ATP biding cassette subfamily B member 11 (*Abcb11*), solute carrier family 10 member 1 (*Slc10a1*), *Slc10a2*, *Slc51a*, and *Slc51b* in the liver and gut. Moreover, the combination of Arazyme and ESLs significantly prevented HFD-induced islet compensation in the pancreas. These results suggest that the incorporation of Arazyme combined with ESLs reduces HFD-induced body weight, hyperglycemia, and hepatic steatosis by regulating liver–gut bile acid circulation in HFD-fed mice. This combination can markedly reduce treatment doses and enhance their therapeutic effects, thereby reducing therapeutic healthcare costs.

## 1. Introduction

The incidence of non-alcoholic fatty liver disease (NAFLD), obesity, and type 2 diabetes is increasing globally. Obesity and type 2 diabetes are important risk factors in the progression of NAFLD [1]. The prevalence of obesity is attributed to obesogenic environmental contributors, including inexpensive and highly processed food and sedentary lifestyles [2]. Additionally, the prevalence of NAFLD is close to 50‒90% in subjects with obesity [3]. Although 60% of liver triglycerides in obese individuals with NAFLD are derived from plasma free fatty acids (which are derived from adipose tissue lipolysis), 26% of liver fat originates from de novo lipogenesis and 15% from dietary fat [4]. In chronic diet-induced obesity and NAFLD, hyperglycemia, hyperinsulinemia, and hyperlipidemia are observed, accompanied by enhanced de novo lipogenesis and cholesterogenesis through the activation of hepatic transcription factors, including sterol-regulatory element-binding transcription factor 1 (SREBP-1), SREBP-2, and MLX-interacting protein-like (MLXIPL, also known as carbohydrate-responsive element-binding protein) [5,6]. A nutrient-rich diet leads to an increase in insulin-induced hepatic phosphatidylinositol 3′-kinase/AKT/mTOR signaling and SREBP-1 expression [7]. Chronic diet-induced obesity and NAFLD-related physiology and pathology are associated with insulin resistance caused by hyperglycemia and hyperlipidemia. Hepatic fatty acids regulate important transcriptional factors controlling hepatic metabolism, including SREBPs, MLXIPL, peroxisome proliferator-activated receptors (PPARs), and forkhead box protein O1 [8].

Lifestyle management with weight loss is the most effective therapy for metabolic-syndrome-related diseases such as NAFLD, obesity, and diabetes. Weight loss of 5% and 7–10% can improve hepatic steatosis and non-alcoholic steatohepatitis, respectively [9]. Although lifestyle management mainly focuses on exercise and a healthy diet, increasing physical activity and reducing the energy content in one’s diet are not always successful in lowering body weight. A plant-based Mediterranean diet is considered one of the healthiest diets, as it aids the prevention of metabolic syndrome with its biologically active compositions, including polyphenols and unsaturated fatty acids [10]. According to the principles of nutritional therapy, a healthy diet focuses on a reduction in saturated fats and carbohydrates. Increasing evidence suggests the significance of phenolic compounds in the management of obesity, diabetes, and cardiovascular diseases [11,12]. Dietary polyphenols, including green tea catechins, resveratrol, and curcumin prevent the development of obesity and obesity-related chronic diseases through regulating the differentiation and proliferation of preadipocytes, fat accumulation (including lipogenesis), fat utilization (including lipolysis, β-oxidation, and thermogenesis), food intake, glucose homeostasis, inflammation, and oxidative stress, as demonstrated in various preclinical and clinical studies [13]. In addition, there has been a growing demand and interest in the bioactivity of insect-based protein-enriched food [14], suggesting that edible insects may have a significant contribution to future food composition.

Arazyme, an approved food additive produced by *Serratia proteamaculans* (*Aranicola proteolyticus*), isolated from the gut of the spider *Nephila clavata* [15], has been reported to have hepatoprotective effects in CCl_4_-treated senescence marker protein-30 (SMP30) knockout mice, inhibiting the FGF-β/Smad pathway and elevating the expression of antioxidant proteins [16]. In chronic high-fat-diet (HFD)-fed NAFLD-like mice, it has been shown to inhibit hepatic lipid accumulation and macrophage-mediated inflammation [17]. Previous studies in animals and humans have shown that a dietary supplement of 70% EtOH extracts of soy leaves (ESLs), containing kaempferol glycosides and pheophorbides, reduces hyperglycemia and hyperlipidemia [18,19]. ESLs exert these effects through improved pancreatic β-cell function and suppression of hepatic lipid accumulation in *db*/*db* obese and diabetic mice [19]. Arazyme and ESLs are both used as ingredients for functional foods in Korea, based on their respective health-promoting functions. The doses of most of the recommended ingredients are too high to demonstrate their benefit effects (e.g., omega-3 fatty acids 1–5 g/day; β-glucan 3 g/day) [20,21], thereby causing some inconveniences in their consumption and industrial application. Hence, in this study, we tried to reduce the doses of ESLs and Arazyme to increase their convenience as ingredients in functional foods. However, the effects of their combined treatment have not been reported. This study evaluated whether the combination treatment of Arazyme with ESLs, at lower doses than the previous effective single dosages, had enhanced effects on body weight gain, hyperglycemia, and hepatic steatosis in HFD-induced obese NAFLD-like mice.

## 2. Materials and Methods

### 2.1. Preparation of ESLs

Yellow soybeans, *Glycine* max (L.) Merr., were cultivated in JeungPyeong County (Chungcheongbuk-do, Korea) for 12 weeks. The dried and ground leaves (150 g) were extracted using 70% ethanol (1.5 L) at 25–30 °C for 48 h. After filtration, the 70% ethanol extract was evaporated to dryness in vacuo to obtain ESLs (30.8 g). The main constituents of the ESLs were kaempferol glycosides and pheophorbides [19].

### 2.2. Animals and Diets

Four-week-old male C57BL/6J mice were housed in a specific pathogen-free facility at the Korea Research Institute of Bioscience and Biotechnology. Mice were kept under controlled humidity (50 ± 5%), temperature (22 ± 2 °C), and lighting (12 h light/dark cycle), with free access to autoclaved water and food. The mice were randomly divided into four groups (*n* = 6 per group): the HFD group, which was fed a 60 kcal% diet (Research Diets, Inc., New Brunswick, NJ, USA) with no supplementation; the HFD+Ara group, fed an HFD with 2 mg/kg Arazyme; the HFD+ESLs group, fed an HFD with 50 mg/kg ESLs; and the combination group, fed an HFD with 2 mg/kg Arazyme and 50 mg/kg ESLs. Arazyme and ESLs were dissolved in sterilized distilled water containing 10% poly(ethylene glycol) and 0.5% Tween-80, and administered daily by oral gavage for 13 weeks.

### 2.3. Measurement of Biochemical Parameters

Body weight was monitored weekly during the experiment. After 9 and 11 weeks of supplementation, mice were subjected to an oral glucose tolerance test (OGTT) and insulin tolerance test (ITT) after the administration of glucose (2 g/kg) or insulin (2 U/kg), respectively. The concentration of blood glucose was measured in tail-vein blood at 0, 15, 30, 45, 60, 90, 120, and 150 min. At the endpoint of the experiment, mice were sacrificed after overnight fasting; the blood from the inferior vena cava was collected in heparin-coated tubes and organs were collected from all mice. Plasma metabolic parameters including fasting glucose, insulin, homeostasis model assessment of insulin resistance (HOMA-IR), glycated hemoglobin (HbA1c), triglyceride (TG), total cholesterol (TC), aspartate transaminase (AST), and alanine transaminase (ALT) levels were analyzed as previously reported [17]. The plasma total bile acid levels were detected using the Bile Acid Assay Kit (Abcam, Cambridge, MA, USA).

### 2.4. Histological Analysis of the White Adipose Tissue, Liver, and Pancreas

The white adipose tissue (WAT), livers, and pancreases were fixed in a 10% formalin solution and processed for paraffin embedding. Samples were cut into 4 μm sections and stained with hematoxylin-eosin (H&E). Images of stained tissue were obtained using an Olympus BX61 microscope system, equipped with an Olympus DP71 digital camera (Tokyo, Japan). The dimensions of the adipocytes and islets were detected by the MetaMorph Image Analysis Software (Molecular Devices, Sunnyvale, CA, USA).

### 2.5. RNA Analysis

The removed liver, ileum, and pancreas tissues were soaked in RNAlater solution (Qiagen, Valencia, CA, USA). Total RNA extraction and cDNA synthesis were then conducted, as previously reported [17]. A PCR system (Applied Biosystems, Forster City, CA, USA) and FastStart Universal SYBR Green Master (Roche Diagnostics, Mannheim, Germany) were used to perform the real-time quantitative RT-PCR. Primer sequences are described in Table S1.

### 2.6. Western Blot Analysis

Protein expression was measured by western blotting analysis using anti-FAS (Cell Signaling Technology, Danvers, MA, USA; #3189, dilution at 1:1000), and anti-SREBP-1 (Santa Cruz Biotechnology, Inc., Dallas, TX, USA; #sc-365513, dilution at 1:500), anti-PPARα (Santa Cruz Biotechnology, Inc., #sc-398394, dilution at 1: 750), anti-UCP2 (Santa Cruz Biotechnology, Inc., #sc-390189, dilution at 1:500), and GAPDH (Bioss Antibodies Inc., Woburn, MA, USA; #bs-2188R, dilution at 1:1000) antibodies. The chemiluminescent signals were developed using HRP Substrate Luminol reagent (Merck Millipore Burlington, MA, USA) and measured by the LAS-4000 Luminescent Image Analyzer (Fuji Photo Film, Tokyo, Japan).

### 2.7. Data Analysis

Results are shown as the mean ± standard error (SE). Statistical differences were analyzed by one-way ANOVA with Tukey’s post-hoc test using JMP software (SAS Institute, Cary, NC, USA). A *p* value < 0.05 was considered statistically significant.

## 3. Results

### 3.1. Combination of Arazyme and ESLs Reduced Body Weight and Adipocyte Hypertrophy

In previous studies, 0.025% Arazyme and 1% ESLs groups were given a supplemented diet, at 12.5 mg/kg Arazyme and 500 mg/kg ESLs, respectively, as effective doses [17]. To assess the combination effect of Arazyme and ESLs, HFD-induced obese C57BL/6J mice were administered daily 2 mg/kg Arazyme, 50 mg/kg ESLs, or a combination of 2 mg/kg Arazyme and 50 mg/kg ESLs by oral gavage, along with HFD supplementation for 13 weeks. The final body, liver, and adipose tissue weights of the mice were compared among groups (Table 1). The initial body weights were similar among all groups. After the 13-week feeding period, the body weight gain and total WAT weight (sum of retroperitoneal, gonadal, and inguinal adipose tissue weights) were similar in HFD+Ara and HFD+ESLs groups compared with the HFD group. However, the combination treatment of Arazyme and ESLs showed a significant decrease in final body weight and weight gain. Food intake was similar among all groups. In addition, liver and total WAT weights in the combination treatment group were 12.3% and 10.7% lower than those in the HFD group, respectively; however, the difference was not significant.

**Figure 1:**
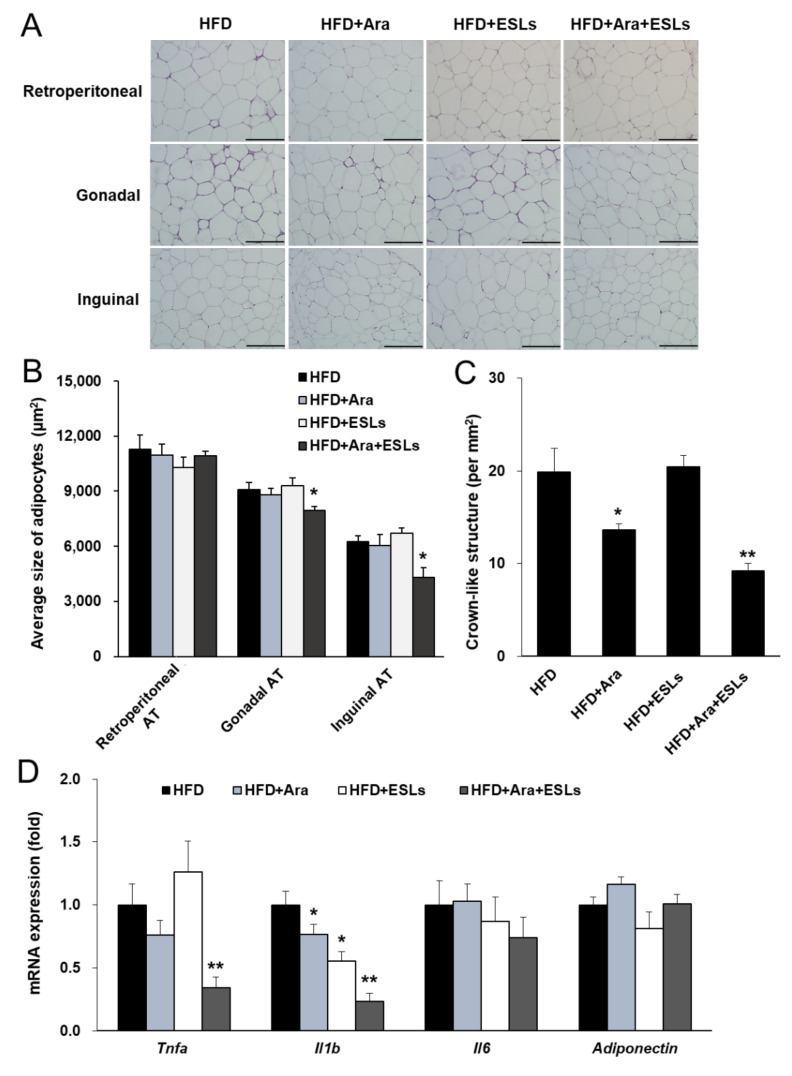
The combination treatment of Arazyme (Ara) and ESLs reduced adipocyte hypertrophy. (**A**) Histology of WAT stained with H&E (×200 magnification, scale bar: 200 μm). (**B**) Quantitative measurement of adipocyte sizes. (**C**) The number of crown-like structures in gonadal AT. (**D**) The mRNA expression levels of gonadal AT were detected by real-time qRT-PCR and normalized using *Gapdh* as a reference gene. Values are presented as means ± SE (*n* = 6). * *p* < 0.05, ** *p* < 0.01 vs. HFD group.

In histological analysis, the mean gonadal and inguinal adipocyte sizes were significantly lower in the combination group than in the HFD group (Figure 1A,B). The number of crown-like structures in gonadal AT decreased more in the HFD+Ara and HFD+Ara+ESLs groups than in the HFD group (Figure 1C). Additionally, the combination treatment group showed a more significant reduction in the number of crown-like structures than the HFD+Ara group. However, the multilocular adipocytes did not appear in the WAT. The inguinal mRNA expression of browning-related factors, including uncoupling protein 1 (*Ucp1*), peroxisome proliferator-activated receptor gamma coactivator 1 alpha (*Pgc1a*), and adrenergic receptor beta 3 (*Adrb3*), was unchanged in all groups (Figure S1). Further, the inflammatory cytokine expression was measured in gonadal AT. The expression of *Tnfa* and *Il1b* in the combination group was lowest among those in all four groups (Figure 1D). Thus, the combination treatment of Arazyme and ESLs significantly reduced HFD-induced weight gain, adipocyte hypertrophy, and inflammatory cytokine expression.

### 3.2. Arazyme and ESLs Supplementation Improved Plasma Glucose and Lipid Homeostasis

After the 13-week feeding of HFD, glucose levels in the HFD+ESLs group were significantly lower (19.1%) than in the HFD group, while no significant changes were observed in the HFD+Ara group; however, the glucose levels were significantly lower (28.4%) in the combination group than in the HFD group (Table 2). The plasma insulin concentration in the combination group was significantly lower (58.2%) than in the HFD group. The index of the HOMA-IR was significantly lower (64.7%) in the combination treatment group than in the HFD group. HbA1c levels were 21.7% lower in the combination group than in the HFD group, although the difference was not significant. Plasma TG levels were unchanged in all groups, but plasma TC levels in the combination group were significantly lower (12.4%) than in the HFD group. The plasma AST and ALT levels, which are indicators of hepatic function, were significantly lower, by 18.5% and 52.1%, respectively, in the Arazyme and ESLs combination group than in the HFD group. In contrast, insulin, HOMA-IR, HbA1c, TG, TC, AST, and ALT levels in the HFD+Ara and HFD+ESLs groups were similar to those in the HFD group.

**Figure 2:**
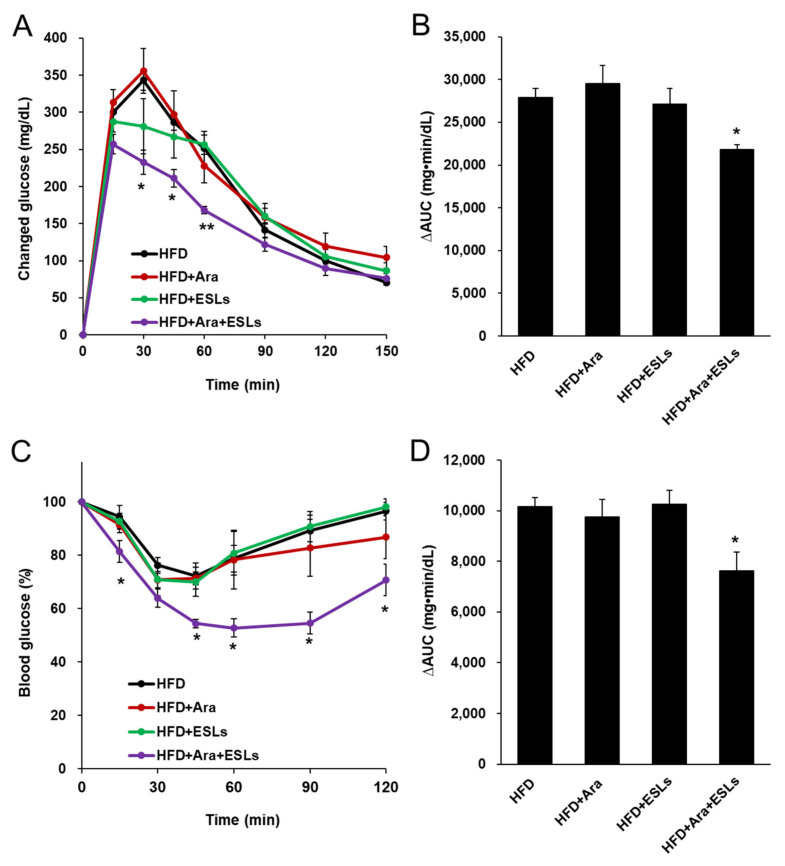
The combination treatment of Arazyme (Ara) and ESLs improved oral glucose tolerance test (OGTT) and insulin tolerance test (ITT). (**A**) The OGTT was performed after oral gavage of glucose (2 g/kg body weight). (**B**) The area under the glucose concentration–time curves (AUCs) of plasma glucose during OGTT. (**C**) The ITT was performed after intraperitoneal injection of insulin (1 U/kg body weight). (**D**) AUCs of plasma glucose during ITT. Values are presented as means ± SE (*n* = 6). * *p* < 0.05, ** *p* < 0.01 vs. HFD group.

Arazyme and ESLs combination treatment significantly lowered glucose levels at 30, 45, and 60 min after gavage with glucose, compared to the other groups (Figure 2A). The area under the glucose concentration–time curve (AUC) was significantly decreased in the Arazyme and ESLs combination group compared to the HFD group (Figure 2B). Mice in the Arazyme and ESLs combination group showed improved insulin sensitivity to glucose levels and AUC after insulin injection compared to those in the HFD group (Figure 2C,D). Collectively, the combination treatment of Arazyme and ESLs exerted a significant effect on the regulation of glucose homeostasis when compared to single treatment groups in the HFD-fed mice.

### 3.3. Arazyme and ESLs Supplementation Reduced HFD-Induced Hepatic Steatosis

**Figure 3:**
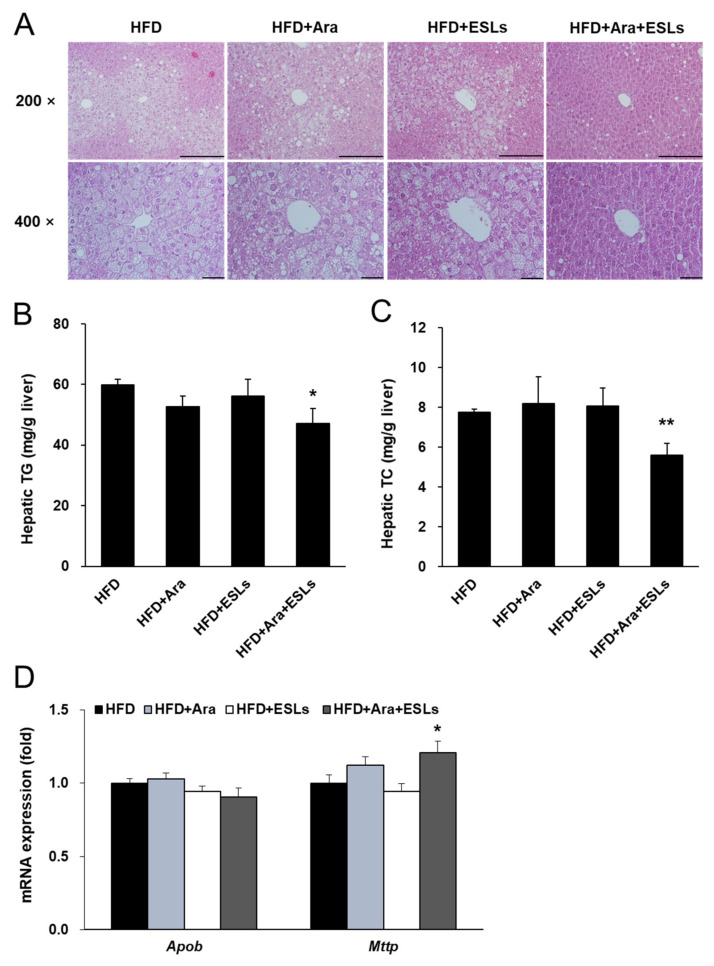
The combination treatment of Arazyme (Ara) and ESLs protected against hepatic steatosis. (**A**) Histology of liver stained with H&E (×200, ×400 magnification, scale bar: 200 μm). (**B**,**C**) Hepatic TG and TC contents. (**D**) TG-secretion-related gene expression in the liver. Values are presented as means ± SE (*n* = 6). * *p* < 0.05, ** *p* < 0.01 vs. HFD group.

Histological analyses were performed to assess HFD-induced hepatic steatosis. Hepatocellular ballooning was markedly noticeable in HFD, HFD+Ara, and HFD+ESLs groups (Figure 3A). In contrast, the combination treatment with Arazyme and ESLs markedly reduced hepatocellular ballooning and prevented the development of hepatic steatosis. In addition, although the hepatic TG content did not change in the groups HFD+Ara and HFD+ESLs (Figure 3B), the combination treatment with Arazyme and ESLs significantly reduced hepatic TG content by 21.0%. Hepatic TC content did not change in HFD+Ara and HFD+ESLs groups (Figure 3C). However, the combination treatment of Arazyme and ESLs significantly reduced TC content by 27.2% compared with the HFD group. Arazyme and ESLs combination treatment significantly reduced lipid-accumulation-related gene expression including *Srebf1*, *Mlxipl*, acetyl-CoA carboxylase 1 (*Acc1*), *Acc2*, fatty acid synthase (*Fas*), stearoyl-CoA desaturase 1 (*Scd1*), and *Scd2*, compared with the single treatment groups (Figure S2). These results were consistent with the previous effects in the individual treatment of Arazyme (12.5 mg/kg) or ESLs (500 mg/kg) as effective dosages. Further, in the combination treatment group, the TG-secretion-related microsomal triglyceride transfer protein (*Mttp*) gene expression was significantly higher in the combination treatment group than in the HFD group, but apolipoprotein B (*Apob*) expression (Figure 3D) did not change.

### 3.4. Arazyme and ESLs Supplementation Enhanced Hepatic β-Oxidation

**Figure 4:**
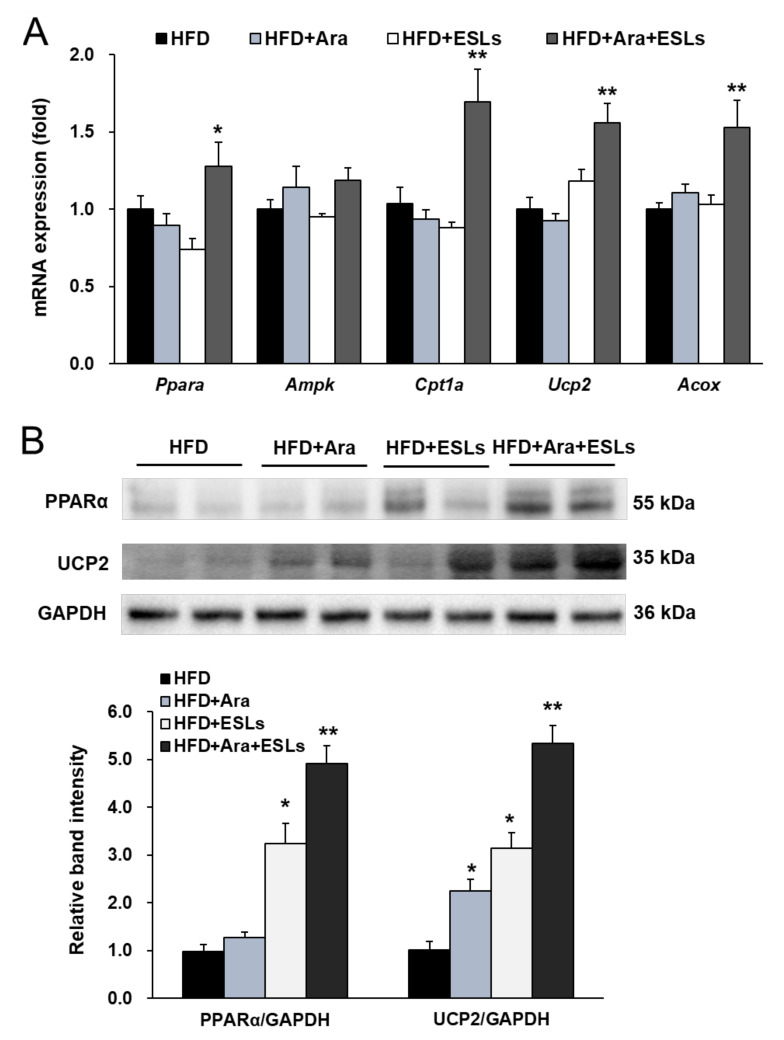
The combination treatment of Arazyme (Ara) and ESLs enhanced hepatic β-oxidation. (**A**) Hepatic mRNA expression levels detected by real-time qRT-PCR and normalized using *Gapdh* as a reference gene. (**B**) Hepatic protein expression levels detected by western blotting with anti-PPARα, anti-UCP2, and anti-GAPDH antibodies. Values are presented as means ± SE (*n* = 6). * *p* < 0.05, ** *p* < 0.01 vs. HFD group.

The combination treatment significantly increased the mRNA expression levels of peroxisome proliferator-activated receptor alpha (*Ppara*) and its target genes, carnitine palmitoyltransferase 1a (*Cpt1a*), *Ucp2*, and acyl-coenzyme A oxidase (*Acox*) (Figure 4A). In addition, the expression of PPARα and UCP2 was markedly higher in the combination treatment group than in each single treatment group (Figure 4B). Taken together, the combination treatment of Arazyme and ESLs significantly protected against HFD-induced hepatic steatosis by suppressing the expression of genes and proteins involved in lipid synthesis and enhancing the expression of those involved in lipid utilization.

### 3.5. Arazyme and ESLs Supplementation Regulated Cholesterol and Bile Acid Metabolism

**Figure 5:**
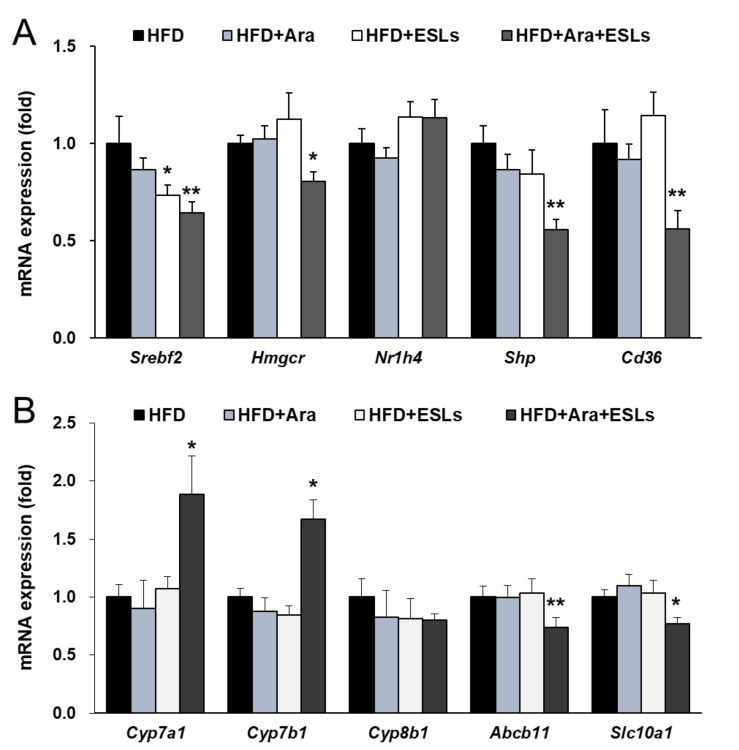

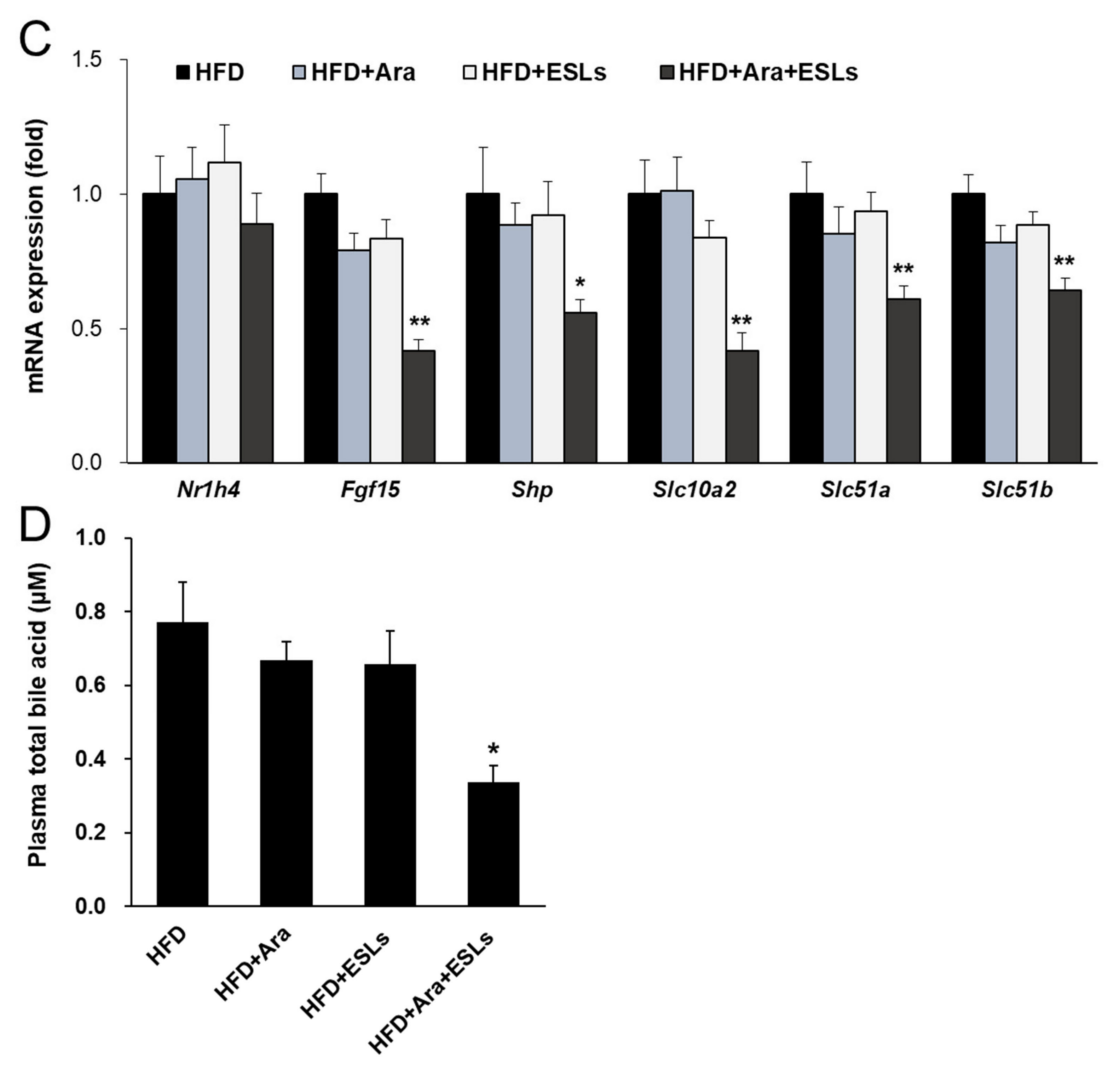
The combination treatment of Arazyme (Ara) and ESLs regulated cholesterol and bile acid metabolism. The hepatic (**A**,**B**) and intestinal (**C**) mRNA expression levels were detected by real-time qRT-PCR and normalized using *Gapdh* as a reference gene. (**D**) The plasma total bile acid levels. Values are presented as means ± SE (*n* = 6). * *p* < 0.05, ** *p* < 0.01 vs. HFD group.

The cholesterol synthesis-related mRNA expressions of *Srebf2* and 3-hydroxy-3-methyl-glutaryl-coenzyme A reductase (*Hmgcr*) were significantly decreased in the combination treatment group compared with the single treatment groups (Figure 5A). Hepatic fatty acid uptake-related expression of the CD36 molecule (*Cd36*) was significantly reduced in the combination treatment group (Figure 5A). However, there were no changes in the nuclear receptor subfamily 1 group H member 4 (*Nr1h4*, also known as farnesoid X receptor) gene expression. The combination treatment of Arazyme and ESLs significantly changed the mRNA expression levels of the genes related to bile acid synthesis, small heterodimer partner (*Shp*), cytochrome P450 family 7 subfamily a member 1 (*Cyp7a1*), and *Cyp7b1*, but not of *Cyp8b1* (Figure 5B). The bile acid transport-related mRNA expressions of ATP-binding cassette subfamily B member 11 (*Abcb11*, also known as the bile salt export pump) and solute carrier family 10 member 1 (*Slc10a1*) significantly decreased in the HFD+Ara+ESLs group (Figure 5B). Furthermore, intestinal fibroblast growth factor 15 (*Fgf15*), *Shp*, *Slc10a2*, *Slc51a*, and *Slc51b* mRNA expressions were significantly reduced in the Arazyme and ESLs combination group (Figure 5C). The plasma total bile acid was markedly decreased in the combination treatment group (Figure 5D). Taken together, the combination treatment of Arazyme and ESLs exerted a significant effect on bile acid metabolism accompanied by the reduction of total bile acid levels and intestinal bile acids reabsorption-related gene expression in HFD-fed mice.

### 3.6. Arazyme and ESLs Supplementation Prevented HFD-Induced Islet Compensation

**Figure 6:**
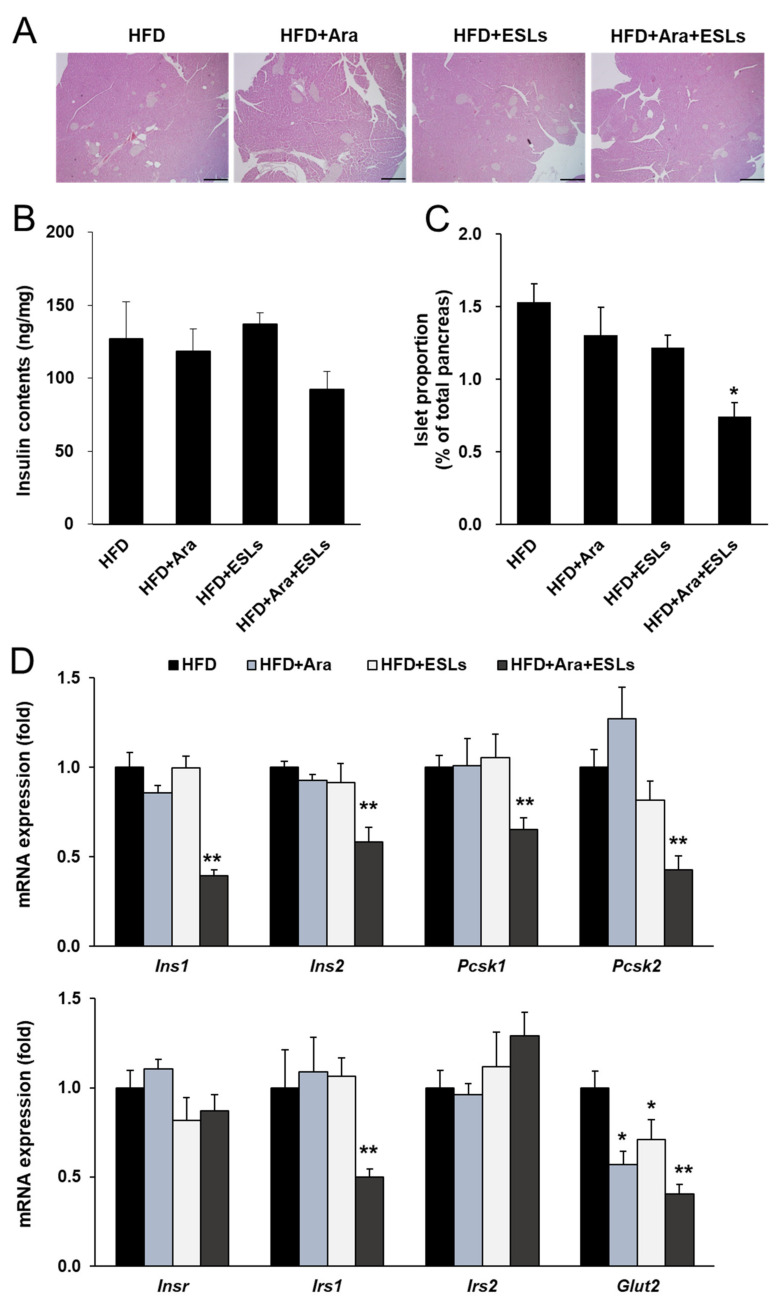
The combination treatment of Arazyme (Ara) and ESLs prevented compensation of pancreatic islets. (**A**) Histology of pancreas stained with H&E (×40 magnification, scale bar: 500 μm). (**B**,**C**) Pancreatic islet proportion and insulin contents. (**D**) Pancreatic mRNA expression levels detected by real-time qRT-PCR and normalized using *Gapdh* as a reference gene. Values are presented as means ± SE (*n* = 6). * *p* < 0.05, ** *p* < 0.01 vs. HFD group.

Histological analysis was performed to assess HFD-induced pancreatic islet compensation. Chronic feeding of an HFD caused pancreatic islet compensation, as demonstrated by an increase in islet size and number. However, the combination treatment of Arazyme and ESLs markedly decreased the islet size and number (Figure 6A). The pancreatic insulin content was markedly lower in the combination treatment group than in the HFD group; however, the difference was not significant (Figure 6B). The islet area proportion, calculated as the total area of the pancreas, significantly decreased in the combination treatment group (Figure 6C). The expression levels of insulin-synthesis-related genes, including insulin I (*Ins1*), *Ins2*, proprotein convertase subtilisin/kexin type 1 (*Pcsk1*), and *Pcsk2*, were significantly lower in the combination treatment group than in the HFD group (Figure 6D). Furthermore, mRNA expression levels of insulin receptor substrate 1 (*Irs1*) and glucose transporter 2 (*Glut2*) in the combination treatment group were the lowest among all experimental groups (Figure 6D). These results indicate that the reduced expression of insulin-synthesis- and β-cell function-related genes may be caused by reduced insulin requirements, which in turn was attributed to improved glucose homeostasis by the combination treatment of Arazyme and ESLs.

## 4. Discussion

Previous studies have shown that Arazyme exerts hepatoprotective effects by improving CCl_4_-induced acute hepatic injury, HFD-induced chronic hepatic injury, and tumor development [16,17,22,23]. In addition, Arazyme treatment inhibits the expression of inflammatory cytokines, chemokines, and reactive oxygen species in lipopolysaccharide-induced human endothelial cells [24]. Dietary Arazyme suppresses hepatic steatosis and steatohepatitis in HFD-induced NAFLD-like mice [17]. The combination treatment of Arazyme with ESLs suppressed hepatic steatosis and fatty acid synthesis in HFD-fed mice. However, in this study, a lower dose of Arazyme (2 mg/kg) was administered compared to that in the previous study (0.025% Arazyme administrated with diet, calculated to be 12.5 mg/kg) [17]. In addition, 500 mg/kg of ESLs improved glucose homeostasis by enhancing pancreatic β-cell function and suppressing hepatic lipid accumulation in the diabetic *db*/*db* mice [19]. In line with the results from the previous study, the combination treatment of Arazyme (2 mg/kg) with ESLs (50 mg/kg) improved hepatic lipid metabolism by suppressing lipid synthesis and enhancing lipid utilization in HFD-fed mice. In addition, the combination treatment suppressed cholesterogenesis, plasma total bile acid levels, and intestinal reabsorption of bile acids. These findings provide evidence of complementary reduction in body weight, glucose, and hepatic TG content following the combination treatment of Arazyme and ESLs. Thus, the Arazyme and ESLs combination makes it possible to reduce the effective doses of Arazyme and ESLs.

Obesity accelerates the process of hepatic steatosis. Hypertrophied adipocytes release free fatty acid through activated lipolysis and cause insulin resistance. The development of hepatic insulin resistance results in the inability of insulin to inhibit gluconeogenesis and an increase in de novo lipogenesis [25]. Hepatic SREBPs, including SREBP-1 and SREBP-2, play critical roles in hepatic lipid metabolism as transcriptional factors [26]. SREBP-1 and MLXIPL, which act as transcriptional factors, can activate hepatic fatty acid synthesis by activating their target genes, which include *Acc1*, *Acc2*, *Fas*, *Scd1*, and *Scd2* [6]. All PPARs act as lipid sensors, induced by nutritional inputs, and regulate inflammatory responses [27]. PPARα, which belongs to the superfamily of nuclear receptors, acts as a major regulator of fatty acid metabolism in the liver by activating fatty acid transport and oxidation [28]. The combination treatment of Arazyme with ESLs significantly improved fatty acid utilization by enhancing PPARα expression. PPARα also activates the over-expression of insulin-induced gene 2a protein, thereby suppressing the function of SREBP-1 in fasting conditions [29]. In patients with NAFLD, PPARα gene expression was decreased in the liver and showed a significant correlation with histological severity [30]. The combination treatment of Arazyme with ESLs significantly reduced HFD-induced hepatic lipid accumulation, which may be caused by reducing *Srebf1*, *Mlxipl*, *Acc1*, *Acc2*, *Fas*, *Scd1*, and *Scd2* expression, and enhancing *Ppara*, *Cpt1a*, *Ucp2*, and *Acox*.

SREBP-2 is the master regulator in hepatic cholesterogenesis and low-density lipoprotein endocytosis [31], and also mediates the output of hepatic cholesterol through the regulation of bile acid biosynthesis [32]. SHP is directly regulated by NR1H4 or protein kinase C zeta; induced SHP suppresses *Cyp7a1* and *Cyp8b1* expression by inhibiting the transcription of liver receptor homolog-1 [33]. The combination of Arazyme with ESLs significantly reduced hepatic steatosis by inhibiting SREBP-1- and MLXIPL-mediated fatty acid synthesis but did not change the plasma TG levels. Consistent with these results, intestine-specific NR1H4 inhibition or liver-specific CYP7A1 inhibition in transgenic mice showed reduced hepatic TG content but did not affect serum TG levels [34,35]. These results might be attributed to the enhanced function of hepatic TG-secretion-related transporters like MTTP. Moreover, inhibited SHP can prevent the development of hepatic steatosis by increasing hepatic very-low-density lipoprotein secretion and by increasing MTTP levels [36]. Intestinal NR1H4 also regulates bile acid metabolism by regulating the reabsorption of bile acid and enterokine FGF15 [37]. Intestinal selective inhibition of NR1H4 reduces HFD-induced hepatic steatosis and obesity with an improvement in metabolic profiles [34,38]. Intestinal Asbt (*Slc10a2*) is the first transporter of bile acids from the intestinal lumen into the ileal enterocytes. Bile acid binds with NR1H4, which activates FGF15 transcription. Enterocyte-released FGF15 and enterocyte-transported bile acid are delivered to the hepatocyte by the portal circulation. Inhibition of intestinal bile acid reabsorption using an Asbt inhibitor restores glucose tolerance, reduces hepatic TG and TC, and improves NAFLD in HFD-fed mice [39]. The combination treatment of Arazyme and ESLs reduced the plasma total bile acid levels by possibly suppressing intestinal bile acid reabsorption and bile-acid-transportation-related gene expression that includes intestinal *Slc10a2*, *Slc51a*, *Slc51b* and hepatic *Abcb11,* and *Slc10a1* genes.

This study had a few limitations. We did not investigate gut function after treatment with Arazyme and ESLs, including nutrient digestion, lipid absorption, and microbiota composition. Improved gut function and the changed composition of microbiota can prevent HFD-induced metabolic mediators. Therefore, further studies are needed to elucidate the in vivo molecular response of Arazyme and ESLs in reducing HFD-induced obesity and NAFLD.

In summary, the present study evaluated the combination effect of Arazyme and ESLs in HFD-induced obese and NAFLD-like mice. The combination treatment with Arazyme and ESLs using non-effective single dosages was capable of enhancing their respective therapeutic effects to suppress HFD-induced obesity, hyperglycemia, inflammatory cytokines, and hepatic steatosis. These results may be attributed to the suppression of SREBP-1-mediated hepatic fatty acid synthesis and SREBP-2-mediated cholesterogenesis, and the enhancement of PPARα-mediated fatty acid utilization by the combination of Arazyme and ESLs. Furthermore, combination treatment of Arazyme with ESLs reduced plasma total bile acid levels and inhibited intestinal reabsorption of bile acid and its transportation. In addition, the combination treatment of Arazyme and ESLs prevented HFD-induced pancreatic islet compensation and β-cell proliferation. These results provide valuable evidence for the preventive and clinical application of Arazyme and ESLs in obesity, diabetes, and fatty liver disease. However, further studies are warranted to elucidate the exact mechanism of action of the combination of Arazyme with ESLs. These findings are of importance as they provide valuable evidence regarding the combination effects of Arazyme and ESLs in enhancing therapeutic effects and lowering treatment doses. Therefore, the present study provides an important basis for lowering the costs in applying these materials in healthcare fields.

## Figures and Tables

**Table 1:** Effects of Arazyme (Ara) and ESLs supplementation on body and organ weights in HFD-fed mice.

	HFD	HFD+Ara	HFD+ESLs	HFD+Ara+ESLs
Body weight				
Initial (g)	20.4 ± 0.6	20.2 ± 0.8	20.9 ± 0.6	20.3 ± 0.5
Final (g)	45.1 ± 1.0 ^‡^	44.1 ± 1.1 ^‡^	45.4 ± 1.5 ^‡^	40.8 ± 1.6 ^‡,^*
Weight gain (g)	24.3 ± 1.0	24.1 ± 0.5	25.0 ± 1.5	20.9 ± 1.6 *
Liver (g)	1.22 ± 0.08	1.17 ± 0.14	1.32 ± 0.13	1.07 ± 0.08
Food intake (g/day)	2.4 ± 0.2	2.4 ± 0.1	2.5 ± 0.2	2.4 ± 0.1
WAT				
Retroperitoneal AT	1.16 ± 0.07	1.17 ± 0.10	1.19 ± 0.09	1.07 ± 0.11
Gonadal AT	2.61 ± 0.16	2.58 ± 0.21	2.58 ± 0.15	2.28 ± 0.15
Inguinal AT	1.95 ± 0.18	1.72 ± 0.35	2.17 ± 0.23	1.45 ± 0.25
Total WAT	5.59 ± 0.32	5.30 ± 0.43	5.86 ± 0.37	4.99 ± 0.35

Values are presented as means ± SE (*n* = 6). ^‡^ *p* < 0.01 compared to the initial body weight in each group; * *p* < 0.05 compared to the HFD group. WAT, white adipose tissue.

**Table 2:** Effects of Arazyme (Ara) and ESLs supplementation on plasma profiles in HFD-fed mice.

	HFD	HFD+Ara	HFD+ESLs	HFD+Ara+ESLs
Glucose (mg/dL)	162.9 ± 11.1 ^‡^	148.0 ± 11.7 ^‡^	131.8 ± 5.1 ^‡,^*	116.7 ± 6.2 ^‡,^**
Insulin (ng/mL)	2.32 ± 0.54	1.76 ± 0.37	2.05 ± 0.59	0.97 ± 0.27 *
HOMA-IR	21.5 ± 6.4	15.6 ± 2.2	17.0 ± 5.2	7.6 ± 2.7 *
HbA1c (%)	6.9 ± 0.5	6.5 ± 0.9	6.3 ± 0.7	5.4 ± 0.1
TG (mg/dL)	146.4 ± 5.2	157.2 ± 11.2	144.8 ± 11.2	141.3 ± 7.5
TC (mg/dL)	211.4 ± 9.1	205.1 ± 2.1	220.4 ± 7.0	185.1 ± 9.6 *
NEFA (mEq/L)	2.25 ± 0.08	2.16 ± 0.11	2.22 ± 0.07	2.35 ± 0.10
AST (IU/L)	67.4 ± 4.4	62.4 ± 2.7	65.0 ± 5.7	54.9 ± 2.5 *
ALT (IU/L)	31.5 ± 3.9	24.8 ± 3.5	29.0 ± 4.6	15.1 ± 0.8 **

Values are presented as means ± SE (*n* = 6). ^‡^ *p* < 0.01 compared to the initial glucose level in each group; * *p* < 0.05, ** *p* < 0.01 compared to the HFD group.

## Data Availability

Data are included in the article or Supplementary Material.

## Supplementary Materials

The following supplementary table and figure are available online at https://www.mdpi.com/article/10.3390/life11070645/s1, Table S1: Sequences of primers used for real-time qRT-PCR, Figure S1: The browning-related gene expression in inguinal adipose tissue. Figure S2: The combination treatment of Arazyme (Ara) and ESLs suppressed hepatic lipogenesis-related gene and protein expression.
