# Supplementary material for: Combination Treatment of Arazyme and Soy Leaf Extract Attenuates Hyperglycemia and Hepatic Steatosis in High-Fat Diet-Fed C57BL/6J Mice
Source: Life (Basel). 2021 Jul 1;11(7):645. doi: 10.3390/life11070645 (PMC8304291; doi:10.3390/life11070645)
Supplement: Supplementary file 1 [file life-11-00645-s001.zip › life-1238326-supplementary.pdf]

Supplementary file

Combination Treatment of Arazyme and Soy Leaf Extract Attenuates Hyperglycemia and Hepatic Steatosis in High-fat Diet-fed C57BL/6J Mice

Hwa Lee [1,‡], Seona Cho [1], Anna Kang [1], Dong-Ha Shin [2], Ho-Yong Park [1,*], and Tae-Sook Jeong [1,*]

[1] Industrial Bio-materials Research Center, Korea Research Institute of Bioscience and Biotechnology, Daejeon 34141, Korea; leehua@kribb.re.kr (H.L.); baby5624@naver.com (S.C.); ank4200@gmail.com (A.K.)
[2] Insect Biotech Co., Ltd., Daejeon 34054, Korea; dhshin@insectbiotech.co.kr
‡ Earlier known as Hua Li
* Correspondence: tsjeong@kribb.re.kr (T.-S.J); hypark@kribb.re.kr (H.-Y.P.); Tel.: +82-42-860-4558 (T.-S.J.); +82-42-860-4650 (H.-Y.P.)

Primer sequences used in this article are described in Table S1.

The inguinal mRNA expression of browning-related factors, including *Ucp1*, *Pgc1a*, and *Adrb3*, were not changed in all groups (Figure S1).

Arazyme and ESL combination treatment significantly reduced lipid accumulation-related gene expression including sterol regulatory element-binding transcription factor 1 (*Srebf1*), MLX-interacting protein-like (*Mlxipl*), acetyl-CoA carboxylase 1 (*Acc1*), *Acc2*, fatty acid synthase (*Fas*), stearoyl-CoA desaturase 1 (*Scd1*), and *Scd2*, compared with the single treatment groups (Figure S2).

Table S1. Sequences of primers used for real-time qRT-PCR

Gene name	Forward primer	Reverse primer
Acc1 (NM_13360)	AGTTTCCCAGCCAGCAGATT	ATCCATCACCACAGCCTTCA
Acc2 (NM_133904)	CCCATCACCACTCCTTCTGA	GTCCGAGTCTCCACAGCAAT
Acox (NM_015729)	GCTGGGCTGAAGGCTTTTACTA	AATCCCACTGCTGTGAGAATAGC
Adiponectin (NM_009605)	CATGCCGAAGATGACGTTAC	CGATACACATAAGCGGCTTC
Adrb3 (NM_013462)	AGGCAACCTGCTGGTAATCA	TCCACAGTTCGCAACCAGTT
Apob (NM_009693)	CCCGTGATACCTACCAGAAT	CTGCCATGTGGTACTTCTGA
Cd36 (NM_007643)	TGCTGGAGCTGTTATTGGTG	CTCAAAGATGGCTCCATTGG
Cpt1a (NM_013495)	CTGCACTCCTGGAAGAAGAA	GTTCTTCGTCTGGCTTGACA
Cyp7a1 (NM_007824)	GACATGGAGAAGGCTAAGAC	TGAAGTCCTCCTTAGCTGTC
Cyp7b1 (NM_007825)	TAGGAGCACATCATCTTGGC	TCGGATGATGCTGGAGTATG
Cyp8b1 (NM_010012)	TCTGGGTCCTCTTATTCCTG	GGTTGAGGAACCGATCATAC
Fas (NM_007988)	TGTGAGTGGTTCAGAGGCAT	TTCTGTAGTGCCAGCAAGCT
Gapdh (NM_001001303)	ACATCATCCCTGCATCCACT	AGATCCACGACGGACACATT
Glut2 (NM_031197)	TTTGTCATCGCCCTCTGCTT	GCAGCGATTTCCTCAAAAGACT
Hmgcr (NM_008255)	TAGGCTTGGTCCTTGTTCAC	GCTTTCTTTGAGGTCACGAC
Il1b (NM_ 008361)	ATGGCAACTGTTCCTGAACTCAACT	ATATTCTGTCCATTGAGGTGGAGAGCT
Ins1 (NM_008386)	ACCCACCCAGGCTTTTGTC	CGGGACTTGGGTGTGTAGAAG
Ins2 (NM_001185083)	CCCCACCCAGGCTTTTGT	GCGGGACATGGGTGTGTAG
Insr (NM_010568)	CTGAACAAAGATGACAACGAGGAA	CTTACAGATGGTTGGGCAAACTT
Irs1 (NM_010570)	GAGAAGAGACTGGCTCGGAAGA	GCCTATTCTGCCCAACTCAACT
Irs2 (NM_001081212)	GGCCCGAACCTCAATAACAA	CCGCGCAACACGAAAAAG
Mlxipl (NM_021455)	CAGATGCGGGACATGTTTGA	AATAAAGGTCGGATGAGGATGCT
Mttp (NM_001355051)	CGTCCCGCTGCTGTCATCTTA	CCCCAATGGACAGCAGGAT
Nr1h4 (NM_009108)	GACAGCGAAGGGCGTGACT	TTTATTTGTGATTTCCTGAGGCATT
Pcsk1 (NM_013628)	CTGCTTTCGCCTTCTTTTGC	CCGCCGCCCATTCATTA
Pcsk2 (NM_008792)	GAAGACGCAGCCTACACCATAA	CTCTCTTTTTACGGTCAAATCCTTCT
Pgc1a (NM_008904)	GTGCAGCCAAGACTCTGTAT	GGTCGCTACACCACTTCAAT
Pparα (NM_011144)	CCTGAACATCGAGTGTCGAA	GTACTGGCATTTGTTCCGGT
Pparg1 (NM_001127330)	ATGTCTCACAATGCCATCAGGTT	GCGGGAAGGACTTTATGTATGAGT
Pparg2 (NM_011146)	TGTCTCACAATGCCATCAGGTT	AGCGGGAAGGACTTTATGTATGAGT
Scd1 (NM_009127)	ACGCCGACCCTCACAATTC	AGTTTTCCGCCCTTCTCTTTG
Scd2 (NM_009128)	CCGTGGCTTCTTTTTCTCTCA	TTCCGCCCTTCTCTTTGACA
Srebf1 (NM_011480)	GAGCGAGCGTTGAACTGTAT	ATGCTGGAGCTGACAGAGAA
Srebf2 (NM_033218)	TCCTCCATCAACGACAAAATCA	ACTTGTGCATCTTGGCATCTGT
Tnfa (NM_ 001278601)	CTCAGATCATCTTCTCAAAATTCGAGTGAC	CTTCACAGAGCAATGACTCCAAAGT
Ucp1 (NM_009463)	CCAGGCTTCCAGTACCATTA	GCCACACCTCCAGTCATTAA
Ucp2 (NM_011671)	GCCTCTACGACTCTGTCAAA	CTTCGACAGTGCTCTGGTAT

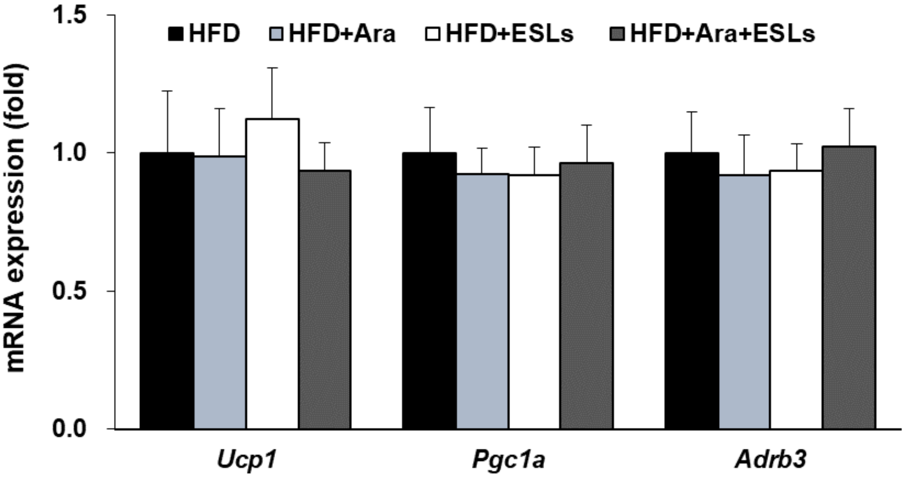

Figure S1. The browning-related gene expression in inguinal adipose tissue. Values are presented as means ± SE (n = 6).

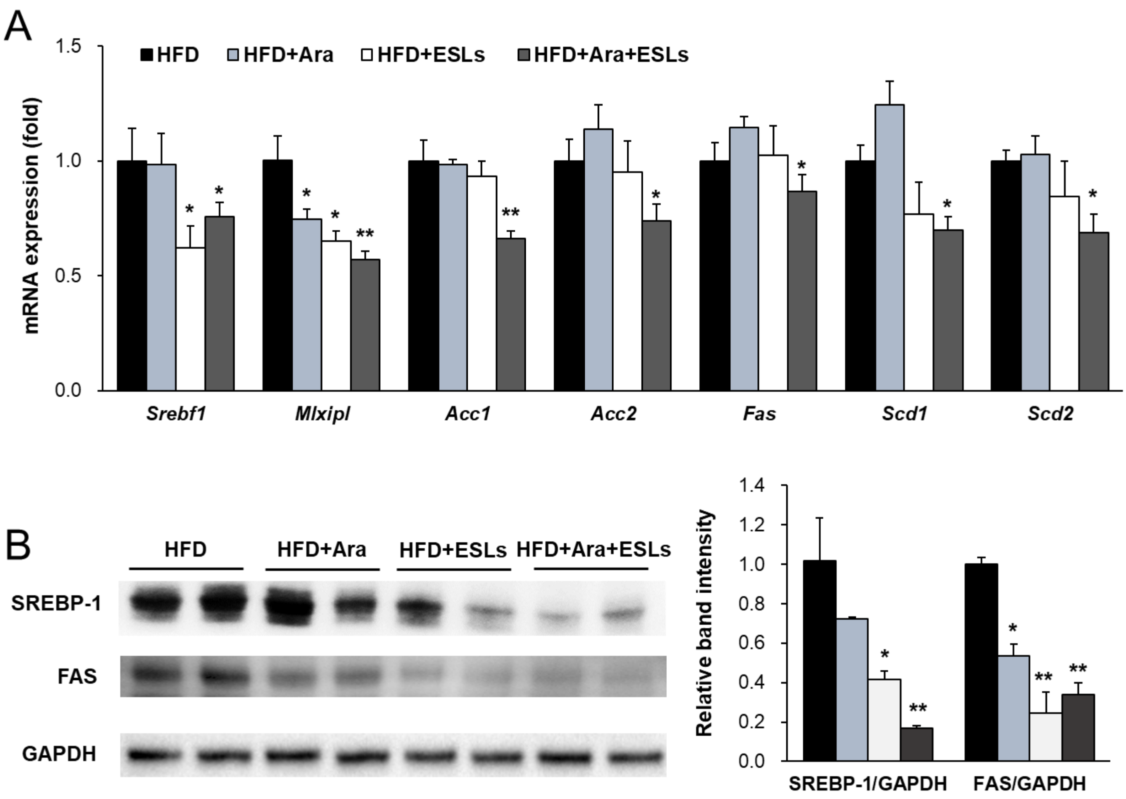

Figure S2. The combination treatment of Arazyme (Ara) and ESL suppressed hepatic lipogenesis-related gene and protein expression. (A) The hepatic mRNA expression levels were detected by real-time qRT-PCR and normalized using *Gapdh* as a reference gene. (B) The hepatic protein expression levels were detected by western blotting with anti-SREBP-1, anti-FAS, and anti-GAPDH antibodies. Values are presented as means ± SE (n = 6). *$P < 0.05$, **$P < 0.01$ vs. HFD group.
